# Compositional Changes in Sediment Microbiota Are Associated with Seasonal Variation of the Water Column in High-Altitude Hyperarid Andean Lake Systems

**DOI:** 10.1128/spectrum.05200-22

**Published:** 2023-04-27

**Authors:** Ignacio Ramos-Tapia, Pamela Salinas, Reynaldo Núñez, Donna Cortez, Jorge Soto, Manuel Paneque

**Affiliations:** a Departamento de Metagenómica, Fundación Bionostra Chile Research, San Miguel, Santiago, Chile; b Laboratory of Bioenergy and Environmental Biotechnology, Department of Environmental Sciences and Natural Resources, Faculty of Agricultural Sciences, University of Chile, La Pintana, Santiago, Chile; Oklahoma State University

**Keywords:** hyperaridity, hypersalinity, metataxonomics, Salar de Atacama, sediment microbiota

## Abstract

The lacustrine systems of La Brava and La Punta, located in the Tilopozo sector in the extreme south of Salar de Atacama, are pristine high-altitude Andean lakes found along the central Andes of South America. This shallow ecosystem suffers from permanent evaporation, leading to falling water levels, causing it to recede or disappear during the dry season. This dynamic causes physicochemical changes in lakes, such as low nutrient availability, pH change, and dissolved metals, which can influence the composition of the microbial community. In this study, we used a metataxonomic approach (16S rRNA hypervariable regions V3 to V4) to characterize the sedimentary microbiota of these lakes. To understand how the water column affects and is structured in the microbiota of these lakes, we combined the analysis of the persistence of the water column through satellite images and physicochemical characterization. Our results show a significant difference in abiotic factors and microbiota composition between La Punta and La Brava lakes. In addition, microbiota analysis revealed compositional changes in the ecological disaggregation (main and isolated bodies) and antagonistic changes in the abundance of certain taxa between lakes. These findings are an invaluable resource for understanding the microbiological diversity of high Andean lakes using a multidisciplinary approach that evaluates the microbiota behavior in response to abiotic factors.

**IMPORTANCE** In this study, we analyzed the persistence of the water column through satellite images and physicochemical characterization to investigate the composition and diversity in High Andean Lake Systems in a hyperarid environment. In addition to the persistence of the water column, this approach can be used to analyze changes in the morphology of saline accumulations and persistence of snow or ice; for example, for establishing variable plant cover over time and evaluating the microbiota associated with soils with seasonal changes in plants. This makes it an ideal approach to search for novel extremophilic microorganisms with unique properties. In our case, it was used to study microorganisms capable of resisting desiccation and water restriction for a considerable period and adapting to survive in ecological niches, such as those with high UV irradiation, extreme drought, and high salt concentration.

## INTRODUCTION

The Salar de Atacama in Chile, part of the Atacama Desert, is considered one of the most arid and ancient regions on the planet (1). The La Brava and La Punta lacustrine systems, located in the Tilopozo sector in the extreme south of the Salar de Atacama, are high-altitude Andean lakes (HAALs) along the central Andes of South America (2, 3). Seasonal and long-term variations in precipitation and evaporation, among other natural factors, generate changes in the behavioral dynamics of the water column in La Brava and La Punta lakes (4, 5), developing areas with greater persistence over time, as well as others with retreat or disappearance during the dry season, called temporary waters (6–8). These dynamics causes physicochemical changes in the water column and superficial sediments, such as low nutrient availability (3), pH change (9), dissolved metals (10, 11), and other factors that can influence the composition and species richness of the microbiota (12, 13). The responses of microorganisms have shown different degrees of resilience to water variation (14). This is relevant for the La Brava and La Punta lakes because of the effects of climate change, which sharpen the precipitation and evaporation cycles in the area (15, 16) and change the dynamics of persistent and temporary waters.

In recent years, several studies have been conducted to investigate the microorganisms that live in this extreme environment and deepen our understanding of the unknown microbial biodiversity present in the Salar de Atacama. Soil (1, 16–18), water (4, 19), and sediment studies (20, 21) all contribute to a better understanding of the microbiota of these extreme environments across locations, diversity, and time. However, in HAALs such as the La Brava and La Punta lacustrine systems, few studies using culture-dependent and -independent methods have reported and identified bacterial phyla such as *Bacteroidetes*, *Haloarchaea*, and *Proteobacteria* (22–25). Furthermore, only a few recent studies on the water microbiota of these lakes have reported interesting findings on how physicochemical parameters modulate the microbiota (4). Herein, we hypothesize that the persistence of the water column over time is associated with changes in the sediment microbiota in the La Punta and La Brava lakes, and these changes are also modulated by abiotic factors.

In this study, we characterized the microbiota present in the sediment of lakes La Punta and La Brava using a metataxonomic approach (16S rRNA hypervariable regions V3 to V4). We combined the analysis of the eco-hydrological dynamics of the lakes, based on the persistence of the water column through satellite images, with physicochemical characterization to determine how the microbiota present in the sediment is modulated in richness and composition.

## RESULTS

Analysis of the water column persistence in the La Punta and La Brava lakes revealed a high degree of seasonal variability due to precipitation and evaporation dynamics (4, 26).

**FIG 1:**
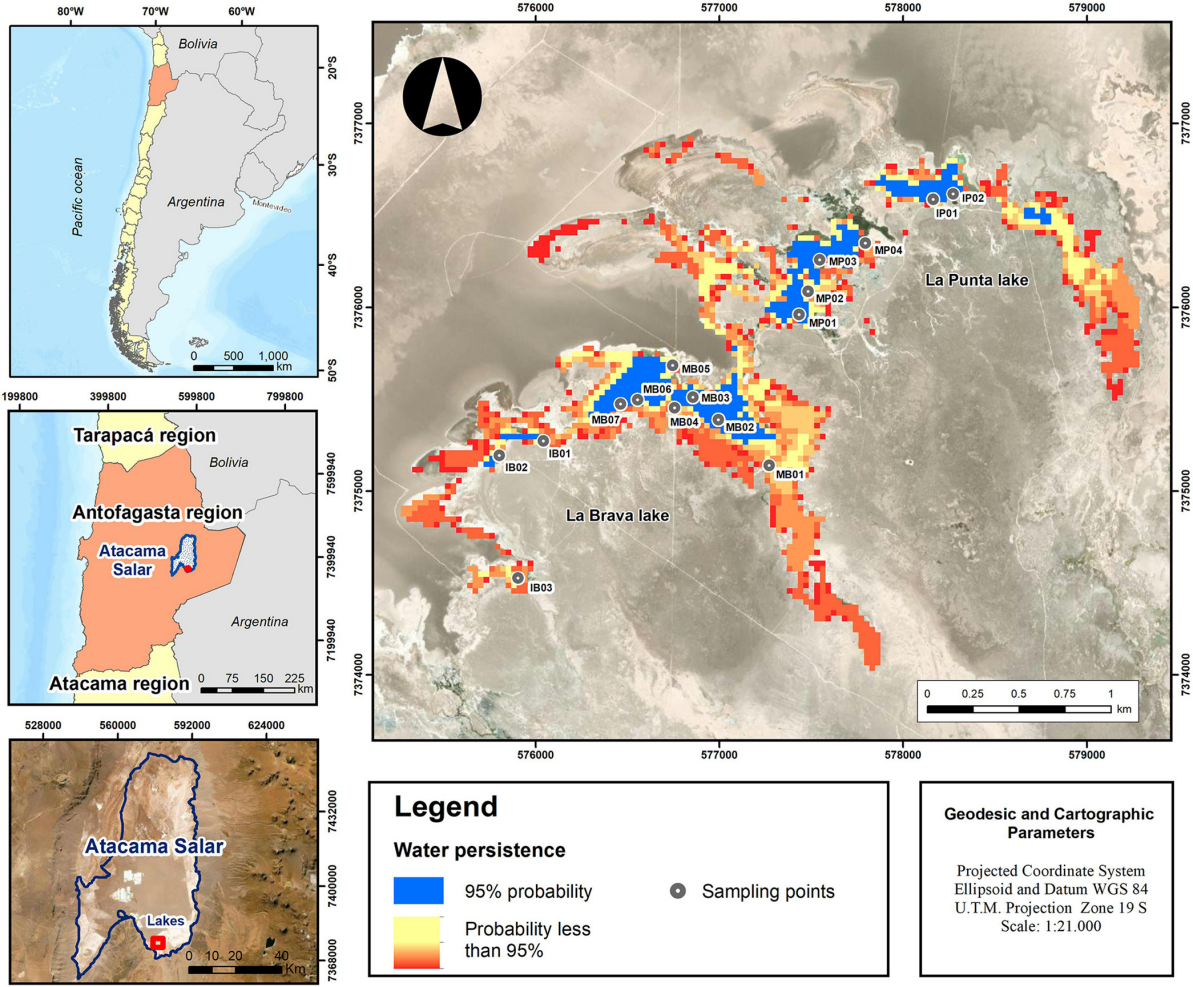
La Punta–La Brava lake system. Distribution of sediment sampling points for La Punta and La Brava lakes in the main bodies with persistent water columns (blue) and isolated bodies with temporary water columns (red).

Only 18.39% of the surface of the La Punta–La Brava system (174.69 ha) had a persistent water column (blue pixels in the cartography), while the remaining surface had a temporary water column between 1 and 10 months of the year (yellow to red pixels; Fig. 1). These ecological dynamics showed that the lacustrine system provided the main body with a persistent water column; therefore, with sediments completely covered by water throughout the year and isolated bodies in the deepest sectors, the water column could be maintained (covered sediments). In other contour sites, the sediment was exposed to wetting and drying conditions throughout the year.

In our study, we collected and measured physicochemical parameters (abiotic factors) that are important and highly relevant in this type of environment. We used the Wilcoxon test to determine if there were any significant differences in pH (*P* < 0.0001), salinity (*P* < 0.01), total silica (*P* < 0.0001), phosphates (*P* < 0.05), DISS Magnesium (*P* < 0.01), hardness (*P* < 0.05), alkalinity carbonates (*P* < 0.01), alkalinity bicarbonate (*P* < 0.001), and total alkalinity (*P* < 0.01) between La Punta and La Brava lakes (Table 1).

**TABLE 1:** Abiotic factor comparison between La Punta and La Brava lakes^*a*^

Variable	La Punta		La Brava		*P*.adj	Significance
	Mean	SD	Mean	SD		
pH	8.21	0.09	7.81	0.21	0	****
DISS oxygen (mg/L)	3.79	0.38	3.38	1.76	0.086	ns
Salinity (g/L)	26.34	10.09	44.64	22.08	0.004	**
Total silica (mg/L)	112.88	11.11	81.48	8.41	0	****
Ammonium (mg/L)	0.09	0.00	0.09	0.00	1	ns
Nitrite (mg/L)	0.01	0.00	0.01	0.00	1	ns
Nitrate (mg/L)	0.5667	0.2643	0.6420	0.3332	0.300	ns
Total nitrogen (mg/L)	2.07	0.97	4.81	8.99	1	ns
Phosphates (mg/L)	0.09	0.00	0.09	0.01	0.047	*
Phosphorus (mg/L)	0.0783	0.0438	0.0930	0.0475	0.190	ns
DISS calcium (mg/L)	341.04	77.28	517.72	245.48	0.056	ns
DISS magnesium (mg/L)	704.47	248.26	1739.70	1172.20	0.001	**
Hardness (mg/L)	3752.6	1212.6	8456.8	5329.1	0.001	**
Alkalinity carbonates (mg/L CaCO3)	26.97	25.02	14.44	29.01	0.008	**
Alkalinity bicarbonates (mg/L HCO3)	377.72	93.17	490.33	129.24	0.001	***
Total alkalinity (mg/L CaCO3)	404.35	73.68	503.96	120.21	0.004	**
TOC (mg/L)	10.69	3.60	10.16	5.16	0.510	ns
Total solids (mg/L)	5.83	2.33	4.90	0.84	0.240	ns

a The column used was a variable between the La Punta (mean and standard deviation) and La Brava (mean and standard deviation) lakes, *P*-value adjusted (*P*.adj), and significance. Wilcoxon test (nonparametric) and the following conventions for symbols indicate statistical significance: ns, *P* > 0.05; *, *P* < 0.05; **, *P* < 0.01; ***, *P* < 0.001; ****, *P* < 0.0001.

There was no significant difference in alpha diversity indices between lakes, ecological disaggregation, or sediment levels (Wilcoxon test, *P* < 0.05). Instead, our sediment samples showed a complex beta diversity structure. Indices differed between the lake variable (all indices, *P* > 0.001), ecological disaggregation (all indices, *P* > 0.001), hydrological dynamics (UniFrac only, *P* = 0.008199), and sediment level (UniFrac only, *P* = 0.0303).

**FIG 2:**
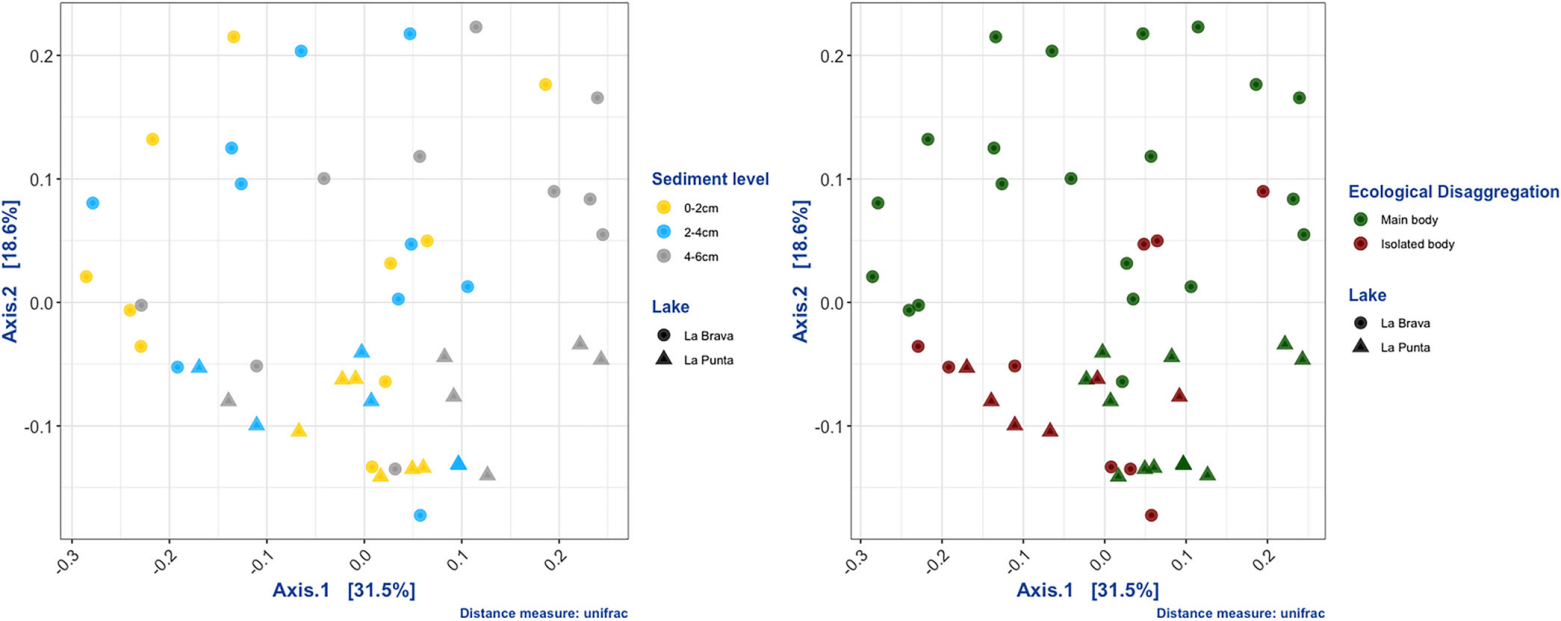
Principal coordinates analysis by the unifrac distance of dissimilarity. Left panel, Lake (PERMANOVA, *P* = 0.00009999), unifrac sediment level (PERMANOVA, *P* = 0.0289), and right panel, unifrac distance in ecological disaggregation (PERMANOVA, *P* = 0.0002).

When we analyzed the lakes separately, only La Punta showed significant differences in ecological disaggregation in the InvSimpson and Shannon indices Wilcoxon test (*P* > 0.001). In contrast, the nonalpha indices were significantly different in Lake La Brava (Fig. 2).

**FIG 3:**
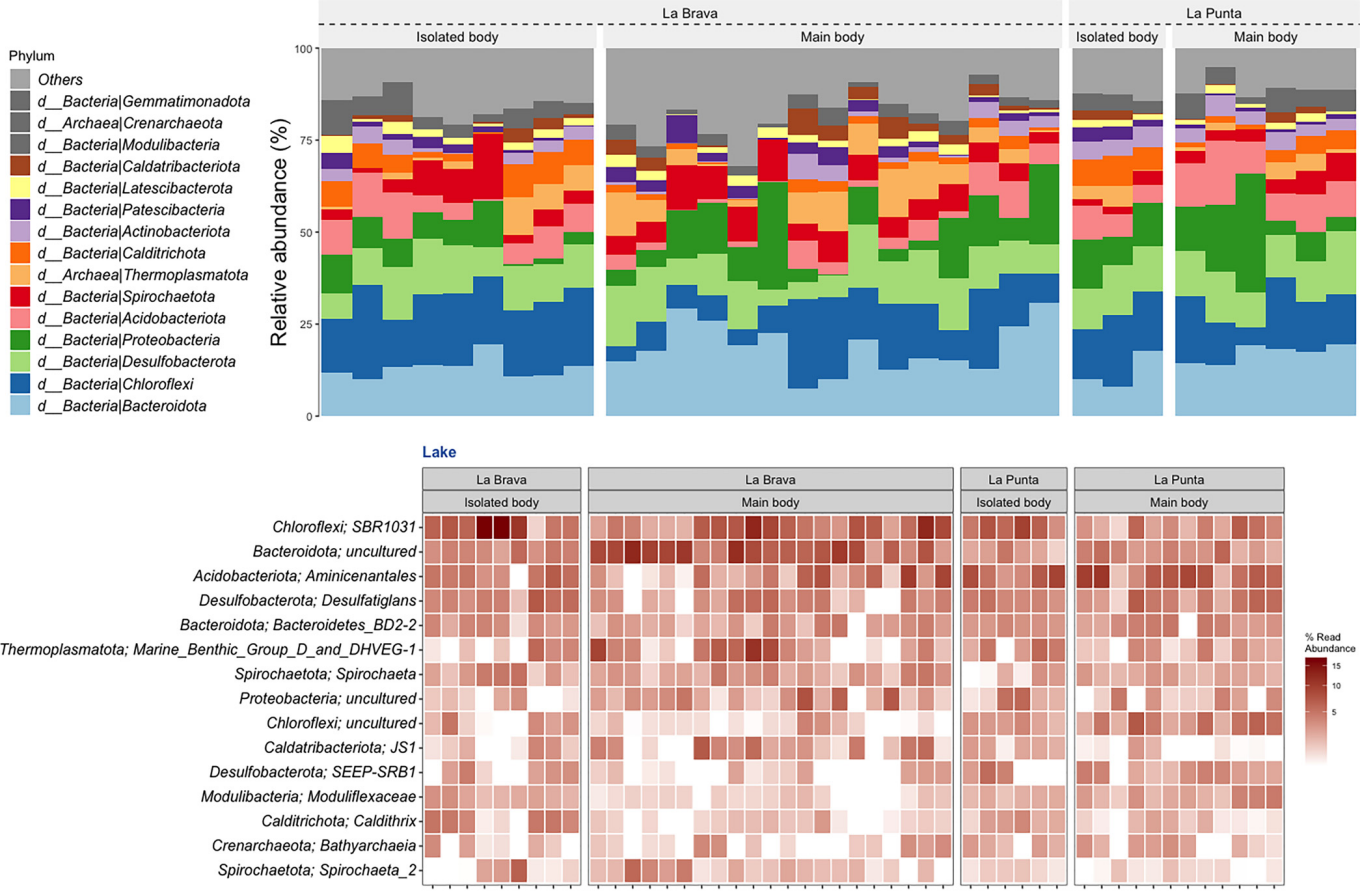
Microbial community composition in La Punta–La Brava lake system. Abundance for lake (La Punta and La Brava) and ecological disaggregation to phylum and genus levels plot top and bottom, respectively.

The composition of microbiota in the La Punta–La Brava lake system is primarily characterized by the phyla *Bacteroidota*, *Chloroflexi*, *Desulfobacterota*, *Proteobacteria*, *Acidobacteriota*, *Spirochaetota*, *Thermoplasmatota* (Archaea), *Calditrichota*, *Patescibacteria*, *Latescibacterota*, *Actinobacteriota*, *Caldatribacteriota*, *Modulibacteria*, *Cranerchaeaota* (Archaea), and *Gemmatimonadota* (Fig. 3, upper panel). At the genus level, we detected *SBR1031*, *Aminicenantales*, *Desulfatiglans*, *Bacteroidetes BD2*, *Marine Bentich Group D*, *DHVEG-*1, *Spirochaeta*, *JS1*, *SEEP*-*SRB1*, *Modulifexaceae*, *Cladithrix*, *Bathyarchaeia*, *Spirochaeta*-2, and unidentified genera (Fig. 3, bottom panel).

**FIG 4:**
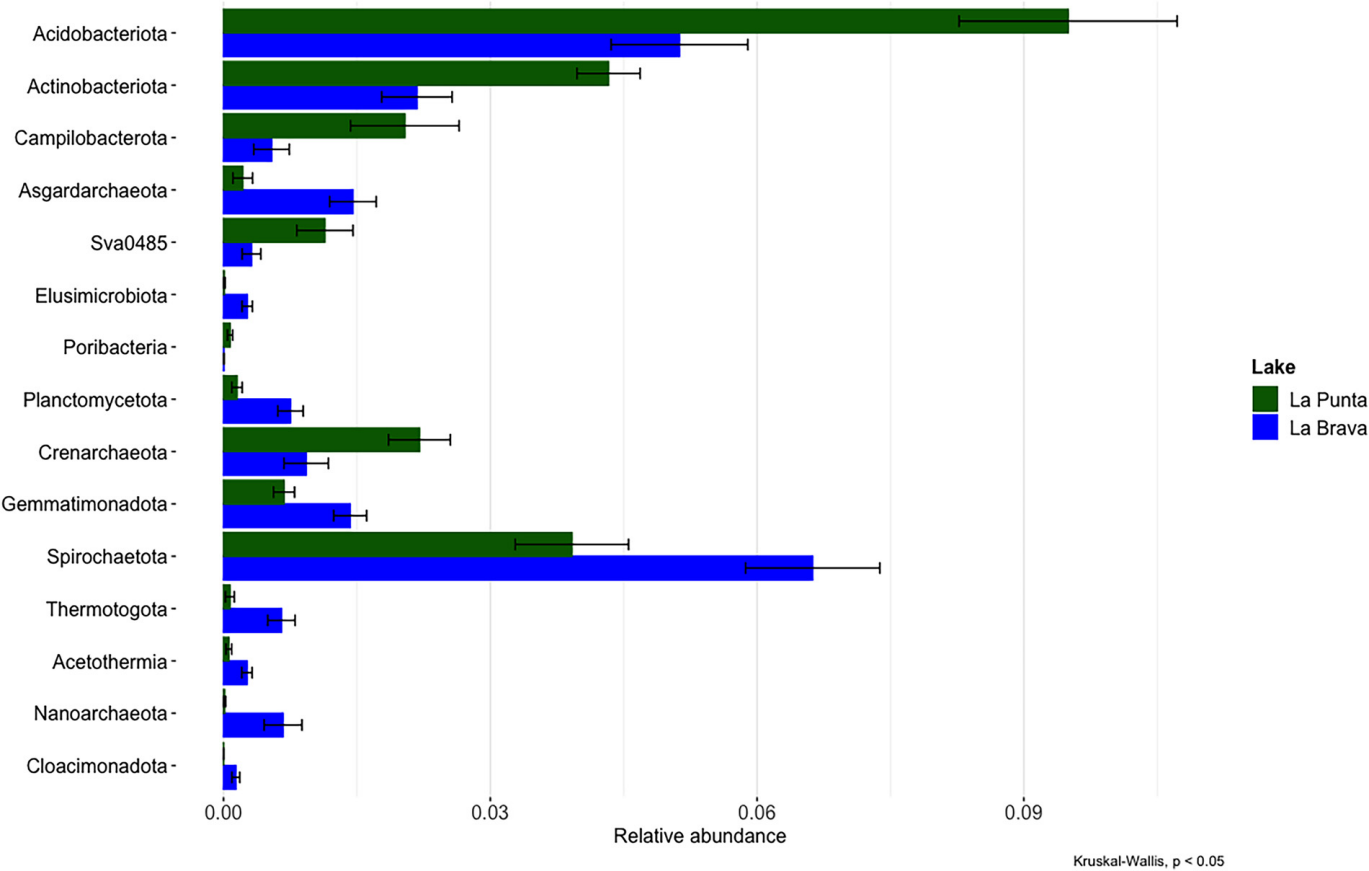
Differential abundance between La Punta and La Brava. Test to phylum level (Krustall Wallis; *P* > 0.05).

We compared the abundance of taxa between La Punta and La Brava (Fig. 4). Certain taxa showed significant differences in abundance between La Punta and La Brava. However, all of these taxa were found in low abundance, between 0.1% and 9%. The taxa with differences at the phylum level are *Acidobacteriota*, *Actinobacteriota*, *Campilobacterota*, *Asgardarchaeota*, *Sva0485*, *Elusimicrobiota*, *Poribacteria*, *Planctomycetota*, *Cranarchaeota*, *Gemmatimonadota*, *Spirochaetota*, *Thermotogota*, *Acetothermia*, *Nanoarchaeota*, and *Cloacimonadota* (Kruskal-Wallis; *P* > 0.05).

**FIG 5:**
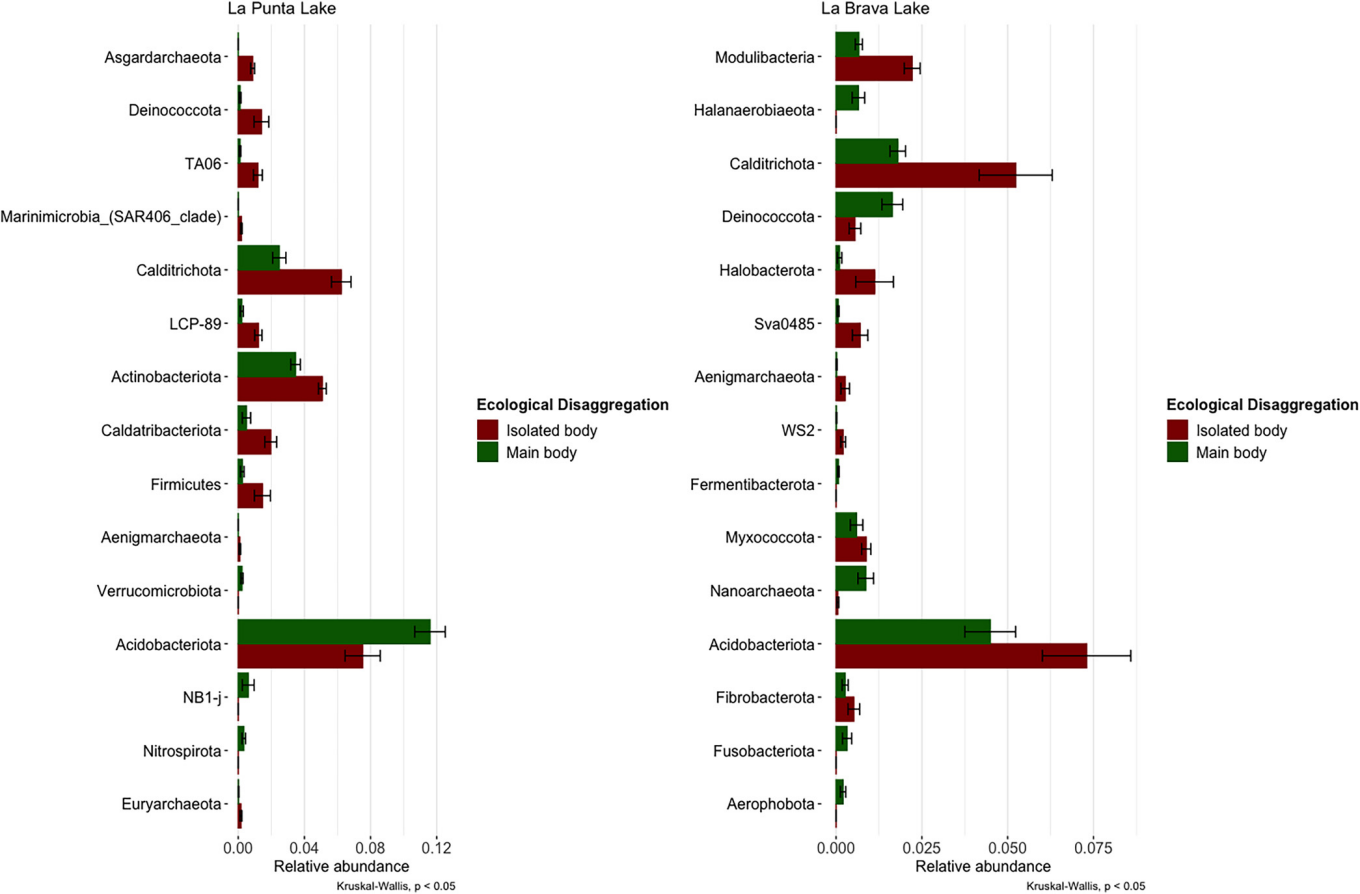
Differential abundance between main and isolated body per lake. The left panel contains abundant taxa, indicating a significant difference between La Punta and La Brava. The right panel shows a significant difference in abundance between the main body and isolated body for each lake. (Krustall Wallis; *P* > 0.05).

Finally, we compared the taxa abundance between the main and isolated bodies of each lake (La Punta and La Brava). Certain taxa showed antagonistic abundances between the isolated and main bodies of each lake (Fig. 5). *Deinococcota* showed abundance in La Punta and La Brava (Kruskal-Wallis; *P* > 0.05). Similarly, the phylum *Acidobacteriota* showed a higher abundance in the main body of La Punta and the isolated body of La Brava (Kruskal-Wallis; *P* > 0.05).

**FIG 6:**
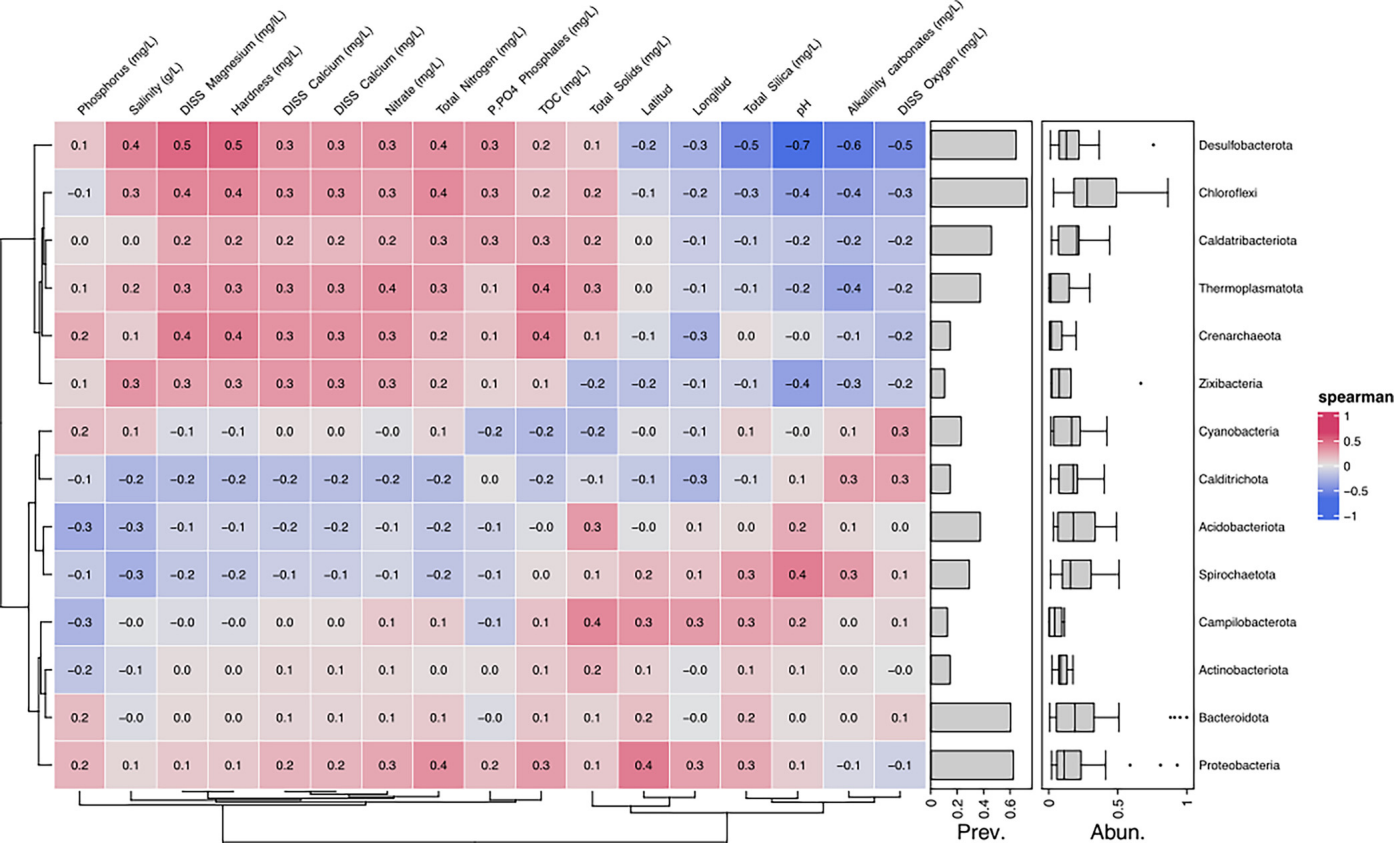
Relations between microbiota and physical-chemical variables. Spearman rank correlation matrix of the bacterial phylum with >1% abundance in at least one sample. Each box shows the Spearman correlation for each taxon (rows) and physicochemical variable (columns). In addition, the prevalence of each taxon and its relative abundance across the samples are added.

The ecological connections and interactions between different genera and physicochemical variables were further investigated using Spearman’s correlation analysis. Fig. 6 shows that the hardness and dissolved magnesium were associated with the influence of the phylum *Desulfobacterota* (Spearman Index 0.5). In addition, pH had the most negative Spearman correlation and was associated with this phylum as well.

Regarding the average correlation of the Spearman index (0.4 and 0.3), the main physicochemical variables that most influence the components of the microbiota are salinity, total nitrogen, dissolved calcium, nitrate, and phosphates, affecting to a greater extent the phyla *Desulfobaterota*, *Chloroflexi*, *Caldatribacterota*, *Thermoplasmatota*, *Crenarchaeota*, and *Zixibacteria*.

Other physicochemical variables influencing other microbiota components include total solids, silica, pH, alkaline carbonates, dissolved oxygen, and longitude/latitude. These variables mainly affected the phyla *Spirochaetota*, *Campylobacterota*, and *Proteobacteria*.

## DISCUSSION

This study focused on characterizing the microbiota of HAALs, with special emphasis on the ecological disaggregation (isolated and main bodies) of each lake and the influence of abiotic factors on the microbial composition of nearby and seasonally connected lakes. This study highlights the importance of microbial diversity in this ecosystem, providing a foundation for understanding how environmental factors in these lakes, including persistent water, affect this diversity and composition.

Seasonal variability in shallow and phreatogenic systems such as La Punta and La Brava lakes is typically related to precipitation behavior, both *in situ* recharges and the activation of surface flows that run off into the lagoons (27–29), even in arid and hyperarid zones (30, 31). This also highlights the effect of evaporation as the main natural discharge of the system (4, 6).

In addition, changes in the morphology of the lagoons inserted in salt flats can be attributed to evaporite growth dynamics, as observed in the La Isla, Agua Amarga, Pajonales, Lastarrias, and Salar Grande lagoon systems, among others (32). Alternatively, as described in the case of the Salar de Llamara, this could be due to derived or accelerated changes in the geological conditions of the lagoons, the quality of the water, and the presence of microbiological structures (5). Authors such as Karaman (33) have also noted that these microbiological structures could influence and be influenced by the hydrological dynamics of lakes by generating structures that segregate or combine sub-bodies of water, thus altering the morphology and surface of the water mirror.

All of the above generate different ecological configurations, such as areas with greater water column persistence and others with important annual oscillations, resulting in temporary aquatic systems (6–8).

Changes in ecological dynamics, in turn, influence ecosystem physicochemical conditions, which determine the composition and species richness of the microbiota (12). Our findings revealed a difference in the persistence of water over time, which we highlighted as the main and isolated bodies (Fig. 1). In ecological terms, this difference in water persistence represents an important selective pressure for the microorganisms that inhabit these regions of each lake, as evidenced by the UniFrac distance in the ecological disaggregation variable (PERMANOVA, *P* = 0.0002) (Fig. 2). Both lakes had distinct microbiota; however, the abundance of taxa varied. In addition, the abiotic (physicochemical) parameters of the two lakes differed statistically. Factors such as salinity (34), pH (9), and dissolved metals (10, 11) can modulate microbiota composition, favoring the growth, prevalence, and abundance of certain taxa (35). Compositional changes caused by physicochemical variations have been described in lagoons present in Salares such as Llamara, which may be faster and more intense in the temporary shallow lagoons owing to seasonality (5).

However, studies conducted in Laguna Turquesa in the Argentine Puna by Villafañe et al. (14) indicated that after degradation processes associated with changes in the physicochemical conditions due to a prolonged water crisis, there was a rapid recovery of microbial systems once the main parameters stabilized. This indicates that the microorganisms are highly resilient.

In our study, the constant variation in moisture availability and the presence of a water column in the sediment of temporarily isolated bodies could indicate that they are microorganisms that are more resistant to environmental variations. In contrast, greater system stability in persistent waters can result in less tolerance to environmental disturbances (12). Authors such as Oehlert et al. (5) reported a high degree of heterogeneity in the microbial communities found in sediments, which respond to different environmental factors, particularly in lacustrine systems with diverse morphologies.

Several studies have shown that archaea are more strongly associated with pH > 8 (36). In our study, the mean pH was 7.96, and we found a limited abundance of archaea in our sediment samples, which is consistent with the pH < 8 samples reported by Santini et al. (36). Similarly, salinity influences microbiota composition. We found significant differences in the salinity concentration between La Punta and La Brava (Wilcoxon, *P* = 0.0041), and these abiotic factors are partially responsible for changes in taxa between lakes (37).

The salinity in La Brava was significantly (40%) higher than that of La Punta. This salinity level would explain the presence of certain phyla related to saline water, such as *Actinobacteria*, which has been found in other studies in saline lakes (38–40). The phylum *Actinobacteria*, in particular, has a high capacity for degrading organic compounds such as cellulose and chitin (41), and many actinobacteria are notable for their ability to produce antibiotic-like compounds (42).

One interesting component of the microbiota composition in the La Punta–La Brava system lake is the presence of Archaea, such as *Thermoplasmatota* and *Crenarchaeota*, belonging to the phylum *Euryarchaeota* (43). This phylum was present in other sediment lake studies in Canada (44) and Switzerland (45) and has been found in previous studies in soils of the same area related to native vegetation (46).

*Thermoplasmatota* is a part of Marine Group II, a clade found mostly in surface seawater; however, certain clades are heterotrophic archaea in deep aphotic waters (47). The genomes collected by this particular group were obtained by metagenomic assembled genomes (MAGs), in part because *Euryarchaeota* has not yet been cultured (48), and our genomes were poorly characterized with the highest number of unknown genes (49).

Order SBR1031, also called *Aggregatilineales*, was one of the taxa with the highest relative abundance. These taxa have been identified using 16S sequences from hot springs (50, 51), contaminated soils (52), and wastewater (53).

Compositionally, microorganisms capable of metabolizing reduced elements may predominate in sectors where anoxic conditions prevail, such as in the deep zones of the sediment covered by a persistent water column. In contrast, aerobic microorganisms would be more prevalent in the thin layer of sediment in contact with the water column or in areas with temporary water columns (12). In our study, we observed significant compositional changes between lakes. We also found differences within lakes between bodies of persistent water (main bodies) and bodies of temporary water (isolated bodies). Certain taxa, such as phyla *Acidobacteria* and *Deinococci* (Fig. 5), showed antagonistic abundance in ecological disaggregation between La Punta and La Brava, likely due to abiotic factors. In genomic and metagenomic studies, the phylum *Acidobacteria* was predicted to possess relevant capabilities, such as using nitrite as an N source (54) and expressing multiple active transporters (55, 56). In the above studies, usable sources of nitrogen, such as nitrite, are present in the La Punta–La Brava lake system (Table 1) and are hypothetically usable by these taxa.

*Deinococci*, a group of bacteria known as *Hadobacteria* (43), have been found in greater abundance in La Punta at the isolated points and La Brava at the main points, indicating that the characteristics of these microorganisms are not out of the range reported in other studies. This phylum is distinguished by being polyextremophile; it includes several species that are resistant to the lethal effect of ionizing radiation and UV light (57) and is also known for its ability to degrade nuclear waste (58). Unfortunately, no study on nuclear waste or similar in the Atacama region has been reported. However, it would be interesting for future studies to delve into the Deinococci group in search of its contribution to and participation in this environment.

This lake system is located in the ecotone between wetland, salt meadow, and dry flat lake ecosystems, and the lakes have a high evaporative rate (26) and subsequently increased water salinity (20, 59). Our results show that different points in each lake have different water persistence, suggesting that desiccation and extreme conditions at these points could considerably affect the microbiota. However, only a longitudinal study at these specific points can indicate which microorganisms inhabit these sectors when conditions are unfavorable.

Regarding microbiota composition, sediment samples differed significantly from water samples from the same site (4). The water samples were dominated by three phyla (*Proteobacteria*, *Bacteroidetes*, and *Actinobacteria*), whereas the sediment samples contained more phyla, including *Chlorofelxi*, *Desulfobacterota*, *Acidobacteriota*, *Spirochaetota*, and archaea such as *Thermoplasmatota* and *Crenaschaeota* (Fig. 3). In comparison with sediment samples from other studies, the composition of the microbiota is similar, including Archaea found in the La Punta–La Brava lake system (40, 44, 45), and is more similar to lakes with a high influence of seawater (60), owing to the high salt content present of the La Punta–La Brava lake system.

The Atacama Desert is an intriguing location for studying microbiota diversity, a specialty in HAALs where characterizations have yet to be fully explored. We found significant differences in the composition of the microbiota of La Punta and La Brava, focusing on the main and isolated bodies, which, based on our analysis of the water column persistence, showed different water persistence over time. The observed variations in soil microbial diversity and composition among plant types can be attributed to the numerous abiotic factors present and measured in these lakes. However, a more in-depth study could reveal how these microorganisms adapt to these conditions. Our results show that both lagoons share a geographic niche. However, the microbiota composition revealed a distinct ecological niche for each lake with differentially abundant microorganisms.

Our limitations in this work lie in the collection time of the satellite images for the detection of the persistence of the water columns, which was limited to the year 2017 and represents a small portion of time. However, new studies obtaining more data (daily or weekly) to process and refine would provide more certainty and sensitivity to the persistence of water columns. In terms of microbiota, owing to intrinsic limitations of the technique, the resolution for the characterization of the microbiota only reached the genus level. A study using complete sequencing of the 16S gene could provide greater resolution of the profile of microorganisms present in this ecosystem. In addition, our approach only provides the composition of the microbiota; an approach using shotgun metagenomics or metatranscriptomic analysis would show possible enriched or transcriptionally active metabolic functions, which are determinants for the survival of microorganisms in this extreme environment.

This study contributes to the comprehensive characterization of the microbiota of the Atacama Desert lakes by providing new information on how physicochemical parameters can modulate the composition and affect the microbiota present in HAALs. In addition, by combining persistence analysis of the water column with satellite images and microbiota analysis, we add a new perspective to correlate the information thoroughly.

This experimental design can be used in the longitudinal analysis of the microbiota for other studies, such as changes in the water level in seacoasts, rivers, or other lakes.

## MATERIALS AND METHODS

### Site of study.

The lagoons La Brava (23° 43′ 44″ S; 68° 14′ 56″ W) and La Punta (23° 43′ 29″ S; 68° 14′ 25″ W) are located 2,305 m above mean sea level at the southern end of the Salar de Atacama in the Antofagasta Region, Chile (Fig. 1). The climate condition is a high-altitude marginal desert, with scarce and variable summer rainfall (4, 26), presenting an accumulated annual average of 19.64 mm year^−1^ (± 19.36 mm), calculated based on the records of the 1975 to 2021 historical series of the Peine meteorological station of the General Directorate of Water (DGA, by its acronym in Spanish). Additionally, the accumulated annual average of evapotranspiration in the area reaches 1,644.79 mm year^−1^ (± 46.92 mm).

From an ecological perspective, these are two shallow, saline lagoons. They are made of a main body of water of a permanent nature; and different temporary sub-bodies, which can dry out depending on the season and rainfall regime. Consequently, the water column and sediment exposure present a high degree of annual and interannual variability (4, 6–8).

In hydrological terms, the lagoons depend in part on the discharge of water from the Monturaqui-Negrillar-Tilopozo aquifer, the depth of the salt wedge at the core of the Salar de Atacama aquifer, and the contribution from lateral sub-basins (4, 61, 62). Additionally, the lagoons respond to precipitation *in situ* and the surface contributions that they activate (sporadic); the main natural discharge is due to the high rate of evapotranspiration (4, 26).

### Sample collection.

Monthly images were downloaded in 2017, belonging to the Operational Land Imager (OLI) sensor on board the Landsat 8 satellite, with a resolution of 30 m. The images were transformed into reflectance and subjected to radiometric (using image metadata in ENVI) and atmospheric corrections (Dark Object Subtraction [-DOS]) (63).

Then, the surface in the presence of water was determined using the AWEInsh index on a monthly scale (64, 65). The cut-off threshold was defined as 0.22 and was determined by photo interpretation (27, 29). This made identifying areas with persistent water columns possible, corresponding to those with permanence ≥ 95% and areas with temporary waters for each lagoon.

Based on the main and temporary bodies of both lakes, 16 sediment collection points were established in the benthic zone, with three replications. They were distributed across 10 collection points in the La Brava lagoon and six points in La Punta (Table 2).

**TABLE 2:** Distribution of collection points and ecological dynamics according to the lake

Sampling point	Coordinates		Lagoon	Ecological dynamics, depending on the persistence of water
	Latitude	Longitude		
MB01	−23.7329	−68.2419	La Brava	Main body - persistent
MB02	−23.7307	−68.2446		
MB03	−23.7296	−68.2459		
MB04	−23.7301	−68.2469		
MB05	−23.7280	−68.2470		
MB06	−23.7297	−68.2489		
MB07	−23.7299	−68.2498		
IB01	−23.7317	−68.2539		Isolated bodies - temporary
IB02	−23.7325	−68.2563		
IB03	−23.7385	−68.2552		
MP01	−23.7255	−68.2403	La Punta	Main body - persistent
MP02	−23.7243	−68.2398		
MP03	−23.7228	−68.2392		
MP04	−23.7219	−68.2368		
IP01	−23.7198	−68.2332		Isolated bodies - temporary
IP02	−23.7195	−68.2321		

The sediment samples were collected using 10 mL sterile syringes, with a tip cut to form a cylinder with a plunger to prevent water entry. Each syringe was submerged in the benthic zone, and approximately 8 mL of sediment was collected. Once the sample was taken from the sediment and separated into layers, the first 2 cm was discarded (the layer at a greater depth of 6 to 8 cm was used to avoid contamination when exposed to the environment).

The remaining sediment is divided into three layers. Layer 1 is the layer in contact with water (0 to 2 cm deep), layer 2 is the intermediate layer (2 to 4 cm deep), and layer 3 is the deepest layer considered in the study (4 to 6 cm deep).

Each sediment layer was placed in a sterile 25 mL falcon tube and labeled with the collection point, layer, and replica. The tubes were immediately closed to avoid environmental contamination and were stored in a refrigerated container at 4°C during transportation to the laboratory (4).

### Physicochemical parameters in sediments samples.

Parallel to the sediment collection, samples were taken from the water columns because their physicochemical properties determine those of the benthic zone to a depth of 10 cm (66). At each sampling point, 1.00 L of water was collected in polypropylene bottles. Immediately, the bottles were closed to avoid environmental contamination and placed in a refrigerated container at 4°C for the duration of transportation to the certified laboratory (4 h).

Total water hardness was determined in the laboratory with other physicochemical parameters, including total organic carbon, orthophosphate, total phosphorus, total nitrogen, nitrite, nitrate, ammonium, total suspended solids, dissolved calcium, dissolved magnesium, total silica, carbonate alkalinity, bicarbonate alkalinity, and alkalinity (67). This procedure was conducted using a commercial service provided by Soluciones en Gestión Ambiental S.A., Santiago, Chile.

The pH, electrical conductivity (EC), and dissolved oxygen (DO) were determined using a HI9829-1 multiparameter meter (Hanna Instruments, Woonsocket, RI, USA). Salinity values (g L^−1^) were calculated according to the conversion method described by Williams (68), using EC values (mS cm^−1^) with an r^2^ of 0.98.

### Extraction and sequencing of DNA.

DNA was extracted from 250 mg of sediment per sample using the DNeasy PowerSoil kit (Qiagen, Hilden, Germany) according to the protocol of the manufacturer and quantified by fluorometry using Qubit 3 equipment (Thermo Fisher) with Qubit 1× dsDNA HS assay kit (Thermo Fisher).

A total of 50 ng of gDNA was fixed to DNA-stable columns (Biomatrica, San Diego, CA, USA) and delivered to Genewiz Inc. (South Plainfield, NJ, USA). For genomic library preparation, a MetaVx kit was used (Genewiz Inc.) and 16S rRNA sequencing was carried out using paired-end MiSeq technology (2 × 250-bp) (Illumina, San Diego, CA, USA). The 16S rRNA amplicons spanning the bacterial and archaeal V3 and V4 hypervariable regions were used to create gDNA libraries. The latter was amplified using forward (CCTACGGRRBGCASCAGKVRVGAAT) and reverse primers (GGACTACNVGGGTWTCTAATCC), similar to those reported by Núñez Salazar et al. (4).

### Microbiota analysis.

The fastq files from the facility were processed in Qiime2 version 2022.11.1 (69). The read length was trimmed forward and reverse sequences to 240 pb using dada2 denoise-paired plugin, and the quality was maintained over PHRED 20. The taxonomic assignment was made with the classify-sklearn plugin using the SILVA database version 138 (68). We used the new unified sequences (OTUs to 99% of identity) to perform alignment using MAFFT (69), the FastTree was used to infer the maximum likelihood, and the phylogenetic tree was from Price et al. (70).

The abundance, taxonomy, phylogeny, and metadata of OTUs were integrated into a phyloseq object for subsequent analyses using the Phyloseq package (71). We used the quality control filters described by Callahan et al. (72): samples of <1,000 reads were excluded, unassigned OTUs were removed, the mean number of reads per taxon was > 1e-5, and OTUs that were not observed more than twice in at least 10% of the samples were excluded.

To compare the physicochemical parameters of the sediment samples, the following convention for symbols indicating statistical significance was used for all tests: ns, *P* > 0.05; *, *P* < 0.05; **, *P* < 0.01; ***, *P* < 0.001; and ****, *P* < 0.0001.

Principal coordinate analysis (PCoA) was used to investigate the Beta-diversity (Jaccard, Bray-Curtis, phylogenetic Unifrac weighted, and unweighted) dissimilarity between samples. Indices were compared using permutational multivariate analysis of variance (adonis) in the vegan R package (73). The models were compared using the Akaike index (74), and the significance was determined using 10,000 permutations.

Visualization of the microbial community was plotted using the R packages microeco v0.11.0 (75) (barplot) and ampvis2 (76) (heatmap). In addition, the differential abundance of taxa was analyzed using the R package microeco, Kruskal-Wallis, and Wilcoxon tests (*P* > 0.05).

For correlation analysis of taxa (phylum level) associated with physicochemical variables, we used the Spearman index in the microViz v0.9.2 package (77).

### Data availability.

All raw sequences were deposited in the National Center for Biotechnology Information (NCBI; Bethesda, MD, USA) database as Bioproject accession number PRJNA603831.
